# Children’s attitudes towards Electronic Gambling Machines: an exploratory qualitative study of children who attend community clubs

**DOI:** 10.1186/s12954-017-0148-z

**Published:** 2017-05-08

**Authors:** Amy Bestman, Samantha Thomas, Melanie Randle, Hannah Pitt

**Affiliations:** 1 Centre for Population Health Research, School of Health and Social Development, Faculty of Health, Deakin University, 221 Burwood Highway, Burwood, Victoria 3125 Australia; 2 School of Management, Operations and Marketing, University of Wollongong, Wollongong, Australia

**Keywords:** Gambling, Gambling venue, Children, Electronic gambling machine, Qualitative research, Social learning theory

## Abstract

**Background:**

This research sought to explore whether children’s visual and auditory exposure to Electronic Gambling Machines (EGMs) in community clubs contributed to shaping their attitudes towards these types of potentially harmful gambling products. This research also examined children’s knowledge of EGM behaviours in adults within their social networks.

**Methods:**

Qualitative interviews were conducted with a convenience sample of 45 children in a regional area of New South Wales, Australia. All children had attended a club that contained gambling products in the previous 12 months. Face to face, semi-structured interviews explored a range of themes including recall of and attitudes towards EGMs. Data were analysed using thematic techniques. Four social learning theory concepts—attentional, retention, reinforcement and reproduction—were used to explore the range of processes that influenced children’s attitudes towards EGMs.

**Results:**

In relation to *attentional* factors, children recalled having seen EGMs in clubs, including where they were located, auditory stimuli and the physical appearance of EGMs. Children also *retained* information about the behaviours associated with gambling on EGMs, most prominently why adults gamble on these machines. Attitudes towards EGMs were *reinforced* by the child’s knowledge of adults EGM behaviours. Some older children’s attitudes were positively reinforced by the perception that profits from the machines would go back to their local sporting teams. Finally, while some children expressed a desire to *reproduce* EGM behaviours when they were older, others were concerned about the negative consequences of engaging in this type of gambling.

**Conclusions:**

Despite policies that try to prevent children’s exposure to EGMs in community venues, children have peripheral exposure to EGMs within these environments. This exposure and children’s awareness of gambling behaviours of adults appear to play a role in shaping their attitudes towards EGMs. While further research should explore the range of other ancillary factors that contribute to children’s knowledge about these machines, policy makers should consider more effective strategies to prevent children from being exposed to EGMs in community venues.

## Background

### Children and gambling: attitudes and consumption behaviours

Youth gambling is a health issue that has gained increasing attention in recent years, particularly given the ubiquitous nature of new gambling technologies and children’s exposure to marketing for gambling products in environments not specifically designed for gambling [1, 2]. Traditionally, research in the area of youth gambling has focused on prevalence [3]. Researchers have demonstrated that children have higher rates of problems with gambling compared to adults [4]. The harms associated with problem gambling for children included mental health issues such as depression, anxiety and low self-esteem, increased engagement with other risky activities and lower educational outcomes [4, 5]. While some research has indicated that some children have a strong intention to gamble both prior to, and when they reach, the legal age [4, 6], there is currently limited qualitative research that examines the factors that influence children’s attitudes and consumption intentions in relation to gambling.

Research that has sought to understand the factors that contribute to children’s gambling attitudes and consumption intentions has primarily focused on the role of three socialising agents: family, peers and the media. For example, researchers have demonstrated that children’s first experiences with gambling are often via their parents [7, 8] and that adolescents who gamble are more likely to have a parent that gambles [4]. However as children get older, peers are more influential when gambling becomes a social activity within friendship groups [4]. Research on the influence of the media has particularly focused around how marketing may shape the perception that gambling is “easy”, "exciting" and “fun” [9]. Studies have identified high recall of marketing strategies amongst children, including gambling sponsorship within sport [2] and the creative strategies and messages within gambling advertisements [10]. What remains unclear is if and how exposure to gambling environments influences children’s attitudes towards gambling.

### Combining family friendly activities and EGMs in community venues

Electronic Gambling Machines (EGMs) (also known as pokies, fruit machines or slots) are recognised as one of the most harmful gambling products in Australia [11], with Australians losing more money on EGMs than any form of gambling [12]. In 2014–2015, over $11 billion was spent on EGMs in hotels and clubs in Australia [12], with problem gamblers accounting for approximately 40% of total EGM losses [13]. There are well-recognised health and social harms associated with EGM use [13], including that the presence of EGMs in communities may increase the prevalence of problem gambling [14]. The majority of EGMs in Australia are located in registered community clubs, with fewer in pubs (hotels) and casinos (the state of Western Australia restricts EGMs from being available outside of the casino) [15].

There has been significant debate about the extent to which clubs, which define themselves as “not-for-profit community-based organisations whose central activity is to provide infrastructure and services for the community” (pg. 3) [16], have become increasingly reliant on EGMs (and problem gamblers) for revenue. Adams (2017) describes the changing nature of the gambling industry, from small cottage industries to a “high-volume consumer enterprise” (pg. 2) [17]. Data suggests that licenced clubs in New South Wales (NSW) derive just under two thirds of their revenue from EGMs [18]. Some argue that EGMs have become "ubiquitous" in clubs and pubs [19] with clubs moving away from the social and community benefits that they once provided [20]. Adams explains the shift from the traditional ‘virtues’ associated with local community venues (as spaces encouraging social activity, moderate levels of gambling and community-based fund-raising) towards products such as EGMs which are “devoid of social engagement or meaning” (pg. 2) and are highly individualised [17]. However, clubs highlight their social benefit for communities [21], including that they play an important role in providing leisure facilities and support for community activities, particularly in regional areas of Australia [22]. For example, community venues have argued successfully for increased numbers of EGMs in their venues, in part based on their commitment to use some EGM funds to provide children’s facilities, such as playgrounds [23, 24]. Furthermore, we have argued in a previous study that the co-location of gambling products within venues that contain family-friendly activities is an ethical issue for policy makers because of the potential for children to be exposed to gambling products, including the role venues may have in normalising gambling for children [25].

### Children’s exposure to EGMs

Concerns have been raised about the potential impact of the co-location of family-friendly and gambling activities on children’s gambling attitudes and consumption intentions [25]. We have argued that the targeting of families with family friendly marketing and activities may encourage families to attend venues that also contain gambling activities and may ultimately expose children to these products [25]. This may arguably contrast with regulations which try to prevent the public (including children) from being unduly exposed to EGMs [26]. While the explicit advertising of EGMs is prohibited in some Australian states [27, 28], the state of NSW requires that the location of EGMs should not “attract the attention of members of the public who are outside the hotel or club premises” (Section 44A) [29]. Furthermore, children are banned from the gaming floor area where EGMs are located and are prohibited from using EGMs until they are 18 years old [29]. However, there is limited research about the extent to which these regulations actually “protect” children from exposure to EGMs. While there have been a small number of studies examining how children interact with low-intensity fruit machines in the UK (which children under 18 are legally allowed to play) [30, 31] , they are not of the same intensity as EGMs in Australia, [32] and so, the applicability of findings in an EGM context is questionable. In 2016, we hypothesised that gambling products may become normalised for children in community venues, particularly if these venues also offer family-friendly products which encourage venue attendance [25]. However, to our knowledge no previous research has comprehensively explored the factors that may shape children’s attitudes and consumption intentions specifically towards EGMs within these spaces.

The aim of the present study was to explore three research questions:

1. To what extent can children recall and describe EGMs and behaviours associated with EGM use in community venues?
2. What factors influence and reinforce children’s perceptions of EGMs in community venues?
3. Do children express current or future consumption intentions to use EGMs?

## Methods

### Methodological and theoretical approach

The research presented in this paper was part of a broader study that qualitatively explored the experiences of families who attended clubs in a regional area of NSW, Australia. We utilised a constructive grounded theoretical approach, as it acknowledges that both researchers and participants create meaning based on past experiences, attitudes and the social constructs with which they live [33]. This approach allows children to describe how they see different phenomena within their environments and has been successfully utilised in other qualitative gambling studies with children [1]. We utilised social learning theory [34] to help to explain the factors that contribute to shaping children’s attitudes towards gambling. This theory outlines how a range of existing social activities, customs and practices are taught to and reinforced by the behaviours of existing members of a social group. A key component of social learning theory is observational learning, whereby individuals acquire symbolic representations of activities based on the modelled behaviour they are exposed to through four sub-processes: (1) *attentional*, whereby individuals recognise features of the behaviour; (2) *retention*, the ability to describe the behaviour following exposure; (3) *reinforcement*, the positive and negative processes that contribute to the behaviour being adopted; and (4) *reproduction*, the ability to replicate the behaviour [34]. The fourth sub-process is difficult to measure in the context of gambling because it is illegal for children to use EGMs. However Bandura (1971) describes that observational learning can occur without specific behaviour replication. To identify this aspect of social learning theory, we explored whether children intended to gamble on EGMs in the future [34]. In gambling, social learning theory has been applied in previous studies to understand how parent and peer behaviour influences gambling consumption intentions in children [4, 35, 36]. However, it has not been used to help to understand if and how different types of community venues (such as clubs) shape gambling attitudes and intentions in children.

### Setting

The study was based in the Illawarra, a regional area of NSW. EGMs have operated in NSW since 1956 [20] and currently have the second highest number of EGMs in the world after Nevada, USA [37]. The Illawarra was selected because of the high concentration of EGMs and high losses on machines in this area [38]. For example in 2014, $143 million was lost on 2614 EGMs in the Wollongong local government area of the Illawarra [38].

### Sampling and recruitment

The sample for the study was family groups comprised of at least one parent and one child (aged 6–16 years old) who had visited a club that contained EGMs, in the past 12 months. We chose a family group model for data collection whereby some interviews included multiple children from the same family [39]. Families were recruited using a range of strategies, starting with convenience and snowball sampling, and then targeted sampling to recruit a range of families with children of different ages, genders and exposure to the club venue. We also utilised social media pages such as local Facebook groups and pages to recruit families into the study. Data collection ceased when we determined that no new data or concepts were emerging from the interviews.

Family groups were reimbursed with a $30 grocery voucher. Participants were contacted through email or by phone and were sent a Participant Information Sheet before agreeing to take part. Parents provided written consent for their child’s participation, and researchers also explained the study to children at the start of the interview and gained verbal consent before the interview began. Ethics approval was obtained from the University Human Research Ethics Committee.

### Data collection

Semi-structured (45–80 min) interviews were conducted between April and October 2016. Interviews took place either in the family’s home or in a public space, such as a cafe or park, and were separated into two parts. First, the parent interview was conducted. While this was being completed, children drew a picture of what they recalled seeing at the club (completed out of hearing distance of the parent). Second, children participated in an interview. Some children were interviewed separately from their parents; however, the parent could sit in on the interview if they or the child preferred. Mindful that the presence of their parent could have potentially influenced children’s answers, we examined the data but identified no clear difference between the responses of children interviewed with their parent and those interviewed on their own. Children were asked a range of questions relating to how often they visited the club, reasons for visiting, activities engaged in while at the club and attitudes towards the club and the gambling products within.

Data presented in this paper is based on a number of tasks performed by child participants. Children often find gambling a difficult concept to talk about, which could partly be due to the fact that it is discussed in schools and family groups less frequently than other public health issues. Consequently, a number of novel data collection techniques (for example, drawing pictures, selecting options from picture boards) were used to enable children to effectively engage with the research. Children contributed to all parts of the interview where they felt comfortable and researchers emphasised there were no right or wrong answers. This was particularly important when multiple children were participating from the one family and they described different aspects of the club. Where there were multiple children from the one family, they could choose whether they completed the interview separately or together. If participating together, the researchers ensured that all children were given each question and the opportunity to respond based on their own experiences. Where possible younger siblings were invited to answer first and notes were taken about the dynamics between siblings.

### Data analysis

Interviews were transcribed from the audio recordings with the permission of participants. Demographic data from participants were analysed using SPSS statistical software. Interview transcripts were uploaded into QSR NVivo 10 and were interpreted using a constant comparative method [40]. This was an ongoing process that occurred during data collection. The data were interpreted using open coding techniques, which involved reading through data and constructing initial concepts as they related to the theory [41]. These were discussed within the research team, and notes were taken regarding the common themes that emerged. As this was a relatively new area of research, additional themes and questions were added to the interview schedule as data collection progressed. The data analysis process was led by the first two authors who met regularly to discuss the study findings. These findings were also discussed with the other co-authors, and feedback was obtained throughout the data collection process. Due to the volume of qualitative data collected, a process of data reduction was employed to develop clearly defined analysis parameters [42]. For this study, this included children’s responses regarding EGMs. Data were categorised into themes based on similar concepts emerging from the data [43]. The data were theoretically grouped based on the four sub-processes in social learning theory [34]. Transcripts were re-read to ensure that theoretical coding was consistent with participant responses.

## Results

### Sample characteristics

The characteristics of the sample are presented in Table 1. Forty five children from 27 families were interviewed. About two thirds of children were male (*n* = 28, 62.2%), with an average age of 11.8 years (SD = 2.7). All children had visited a club in the year prior to the study, with almost half reporting they had visited clubs at least once a month (*n* = 19, 42.2%). One in five children attended clubs at least once a fortnight (*n* = 9, 20.0%). Just under half reported knowing an adult who gambled on EGMs (*n* = 21, 46.7%) and just over half indicated they would like to try gambling on EGMs when they were older (*n* = 23, 51.1%).

**Table 1:** Sample characteristics

Gender	
Male	28 (62.2%)
Female	17 (37.8%)
Age	
6–8	6 (13.3%)
9–11	11 (24.4%)
12–16	28 (62.2%)
Club visit frequency	
Once per week	6 (13.3%)
Once a fortnight	3 (6.7%)
Once a month	10 (22.2%)
Once every 2–3 months	7 (15.6%)
<3 times per year	19 (42.2%)

### Attentional: recognition of EGMs in the venue and knowledge regarding product use

The first organising theme related to the attentional factors that created awareness of EGMs within clubs. This included children’s recall of EGMs in the venue, and the ability to describe EGMs, including their appearance and auditory stimuli (for example, hearing the sounds from machines). When prompted, all children had an awareness of EGMs, with the majority able to specifically recall seeing EGMs in a venue (*n* = 44, 97.8%). Some described the location of EGMs in the venue, for example, that EGMs were located close to the entrance of the club, and they had seen EGMs “as soon as you walk in”. Others described seeing EGMs as they moved through the club to the restaurant; “you can walk through and you can see them”, with one 12-year-old boy stating that he would “get a little glimpse” of the machines.

Some children were aware that EGMs were in spaces where children were not allowed to go, with one 13 year old describing a barrier with “little lines” through which he could “hear and see the pokies”. Several children described that while they could not see EGMs due to screens or frosted glass doors, they knew they were there. Children’s exposure to EGMs was not confined to inside venues, with two boys from the same family describing that they saw EGMs through club windows as they drove past on their way to the supermarket.

The majority of children could provide descriptions of EGMs. While most related to how the machines worked or why adults use them, some children provided detailed descriptions of their physical appearance. Children described “bright lights” or “colourful colours”, while a 14-year-old boy recalled that EGMs “have money signs”. Older children provided more detailed descriptions, describing buttons and symbols on the machines and the patterns they thought were needed to win:

*“It’s like just a rectangle box and then you’ve got like probably a button and then the only one I can think of now is where three things rotate. And you’ve got different numbers; I think seven’s the best. And they’ve got different pictures and they’ve all got to line up*.”—Male, 14 years, attended club monthly.

Children very rarely used gambling terms to describe machines. Rather, they used softer terms such as “play” or referred to EGMs as a “game”. Many children perceived that EGMs were games whereby adults could “win” money. One 12-year-old boy referred to an EGM as an “arcade game”, while an 8-year-old boy explained that the machine contained money that you could win:

*“They’ve got money in them. So you can win money from them.”*—Male, 8 years, attended club weekly.

Several boys and children over 12 were able to provide specific descriptions of how to use EGMs. For example, they explained the process of “putting money in” to the machines, described the spinning reels or numbers lining up. A 12-year-old boy explained that you could win “free things” and win the “jackpot”. Children also made physical gestures such as pulling down a lever on the side of the machine, or “pressing a button” to make the reels spin.

*“You put money into them and pull a lever and get money if you get lucky”—*Female, 12 years, attended club every 2–3 months.

Some children reported seeing EGMs in environments other than clubs, including in movies or on television, and recognised the EGMs in the club based on their prior knowledge.

Some children, predominantly boys, described the sounds made by EGMs. Many reported hearing the sounds of EGMs in the club while eating dinner or walking through the club to get to the restaurant. They had heard the machines play music and described other sounds such as “balls rattling around”, “coins dropping” or “banging and clanging”. During the interviews, many children replicated the sounds of EGMs, such as “bing” or “ti-ting”. This use of onomatopoeic language was particularly prominent when children were describing the positive sounds EGMs made when someone won. For example, one boy aged 12 said a winning EGM made a “bing, bing, bing, bing!” sound. He went on to describe what happened when he had heard people winning on EGMs in the club:

*“You hear the money hitting the metal and then you hear, ‘Ding, ding, ding, ding’. Then the big winner sign lights up on the top of the pokie machine and everybody screams, ‘GET THE MONEY!’* ”—Male, 12 years, attended club less than three times per year. Some children described how the sounds from the EGMs were the loudest sound that they could hear when in the restaurant, with one girl stating:

“*I don’t take much notice of them, they’re just there - I block it out*.”—Female, 16 years, attended club weekly.

### Retention: recognising and describing adult behaviours associated with EGM use

The second organising theme related to children’s ability to describe adult behaviours associated with EGM use (retention). This included gambling on EGMs, most prominently relating to why adults gamble on these machines and the benefits and harms associated with EGM use. The primary reason for EGM gambling was the perceived chance of winning money. Children had an inflated perception of the ease of winning money from EGMs. For some children (even those who went on to describe harms), winning was described as a certainty. For example, a 14-year-old boy described EGMs as being used by adults as a way to “earn money”, while a 10 year old stated that EGMs were used to “give the family money”. The same boy described how wanting to provide money and to give a good news story to your family could be one reason why individuals become addicted to EGMs. Talking to his older brothers, who were also involved in the interview, he stated:

“*You’re trying to win. You never know if you could win or not. And you want to win! If you win you can go home to your children and say, ‘Oh, look what I won on the pokies. You might be able to do this when you’re older’.* ”—Male, 10 years, attended club less than three times per year.

Concepts associated with luck and fun were also used to describe why adults used EGMs. Some children referred to EGM returns as prizes that adults wanted to win. Several children perceived that adults used EGMs because they “might get lucky” or were “hopeful that they will get lucky”. Other positive reasons included adults thinking EGMs were fun, for entertainment, for social reasons or to relax:

“*It’s a form of entertainment, it’s sort of a little bit of pleasure and a bit of adrenaline that sort of ‘what’s it going to be?’* ”*—*Female, 14 years, attended club fortnightly.

Some children over 12 years old, believed adults used EGMs to escape, if they were not happy or “if they have nothing to do”, while others stated that people might gamble on EGMs if they were “stressed out with life”.

However, along with the positive aspects of machines, children also described the negative consequences of gambling on EGMs. For example, the words “addicted” or “addiction” were used by older children to describe consequences. Other children, including young children, were able to articulate (without prompting) the broader social harms associated with financial losses. Some discussed financial implications of EGM gambling, such as not being able to afford rent or finding it “hard to buy food and keep your kids not hungry”. Some also identified emotional responses that individuals might have if they had developed a problem with EGMs, including that people would get angry or “rage”.

Some children had strong negative reactions to EGMs, even if they did not personally know someone who gambled. One 12 year old girl, who did not know an adult who gambled described that she “hated” gambling. However, her anger was directed to the irresponsibility of people who spent money that they were unable to afford on gambling:

“*People go broke and then ask for money and then as soon as they get money they just go and spend it on the pokies…I think it’s stupid.*”—Female, 12 years, attended club fortnightly.

A few children were able to clearly articulate where they had learnt about the harms associated with EGMs. A small number recalled seeing media stories about professional sports people (predominantly from the National Rugby League) who had experienced problems with gambling and who had “lost all their money from it”. One 12-year-old girl drew on her personal experiences of seeing adults who gambled on EGMs at her local club, who she described as “zombies” with “red eyes”.

### Reinforcement: factors that influence children’s perceptions of EGMs

The third organising theme related to factors that reinforced children’s perceptions of EGMs. This included the role of adults around them who used EGMs and the benefits associated with EGMs. Just over one third of children reported knowing adults (predominantly family members and family friends who they visited the club with) who used EGMs. Some children described their parents’ use of EGMs, with one child stating that her parent did not use the machines when the child was with them at the club, but that she knew about EGMs through her mother’s discussions with others. Many descriptions related to parent’s financial wins and losses, with children commonly describing only small losses but big wins.

*“*[Mum] *put $50 in and she came home with no money and the next day she came here and put $25 in and bang, smack, came home with $1,000”*—Male, 10 years, attended club less than three times per year.

Children were often careful to point out that the adults they knew were responsible with their gambling. For example, they described that adults they knew were “not gambling a lot” or “setting limits”. Several children described individuals they knew as only using EGMs on “special occasions”. One boy aged 16 years said he had “heard stories of [individuals] winning money and stuff” from his older sisters’ friends. This boy also worked at a local fast food restaurant and had recently attended a work meeting at the local club where some individuals used EGMs.

“*Everyone that I know, doesn’t put that much in, and they don’t do it very often. It’s kind of just like a one-off thing.*”—Male, 16 years, attended club less than three times per year.

Finally, the majority of children referred to the club’s role in the provision of EGMs. Some children had formed a positive attitude towards EGMs based on perceptions that the club gave money from EGMs back to the community. Some children, particularly those who played for a club-sponsored sporting team, recalled examples of receiving benefits from the club such as uniforms, discounted sporting memberships and food vouchers. Others attended events such as dance concerts or sporting presentations within the club venue. Some children expressed a view that in order for the club to support the community, people had to lose money. They not only believed that the presence of EGMs was a good thing for the club “because they’re getting money” but also acknowledged “for people who are using them and losing their money, that’s not good.” A few children were conflicted by the perceived benefit the club would receive from individuals who lost money on EGMs.

“*It’s not harming, it’s just mean because it takes your money and it’s not fair for you. But it is a bit fair because they buy equipment for you to keep you safe and to keep you comfortable and to keep you entertained in the clubs.”*—Male, 10 years, attended club less than three times per year.

### Reproduction: factors that influence children’s desire to use EGMs

The final theme related to children’s desire to replicate or *reproduce* behaviours relating to EGMs. This included a desire to use EGMs now if they were allowed, the influence of gaming products within the venue and their future consumption intentions. While most children stated they would not gamble on EGMs while under 18 years old, many found it difficult to conceptualise what 'legal' gambling was or what they would do when they were older. Children often said that allowing children to gamble would be “irresponsible” and that “they might get addicted at a small age”. Some children stated that they would not gamble because they were fearful of EGMs and, in particular, losing money. For example “I don’t want to be one of those poor people” or “I don’t want to waste my money”.

A few children reported a current intention to use EGMs before they turned 18 because they had “games on them” or because they enjoyed playing the non-gambling arcade games at the club. Children who played arcade games at the club also more commonly described wanting to try EGMs when they were older. For example, an 8-year-old boy said he would use EGMs now because he perceived he always won at the “claw” arcade game (whereby a claw is used to pick up chocolates inside a glass box).

The majority of children who said they would use EGMs as adults also knew an adult, usually a parent or grandparent, who used EGMs at the club. These children often gave examples of adults winning money on EGMs. A few children described that they would try EGMs because they perceived they were fun. For example, a 16 year old stated that he would use EGMs when he turned 18 because “they seem pretty fun”. However, narratives of responsibility also were evident in children’s responses. For example, some children described placing limits on their gambling behaviours, stating that they would use EGMs as adults, but “not excessively”. Other children stated an intention to try EGMs either as children or adults but said that they would not gamble regularly. For example, one child stated that he would “give it a go” but would not “waste my life on it”, while a 12 year old stated he would be interested in trying EGMs “just once” but that his father would have to operate the machine to “press the button to see what I get”.

## Discussion

This study aimed to explore the extent to which children can recall and describe EGMs and behaviours associated with EGM use in community venues; the factors that may influence and reinforce children’s perceptions of EGMs in community venues; and finally, whether children indicate current or future consumption intentions towards EGMs. Findings present three key areas for discussion relating to the factors that influence children’s attitudes and future consumption intentions of EGMs.

The first is the *attentional* factors identified in this study. The descriptions children provided about EGMs, including their recall of the machines within venues, and specific factors associated with the machines such as winning sounds, highlight children’s peripheral exposure to EGMs within the club environment. While we acknowledge that there may be factors outside of the venue that influence children’s knowledge about EGMs, children were able to describe exposure specific to club environments. As such, we would argue that current regulations are not effective in creating environments which completely protect children from being exposed to EGMs. This study raises questions about how policy makers define *exposure*, and that we cannot assume that just because children are not physically entering the gaming room or sitting at a machine pushing buttons, they will not be exposed in some way to these products. Despite state regulations designed to prevent the promotion of EGMs and prohibit young people from using EGMs [29], this study shows that children recall the visual (flashing lights) and auditory (winning sounds) aspects of EGMs in the venues. These visual and auditory stimuli may contribute to positive perceptions amongst some children about EGMs which are associated with winning money. Research has previously described how the audio cues associated with EGMs promote gambling as a fun activity, suggest the likelihood of big wins, and promote winning as significantly more likely than losing [44], and that audio cues specifically related to winning have a significant impact on reinforcing adult gambling behaviours [45–47]. We would recommend that policy level consideration should be given to measures that ensure children are not exposed to such auditory stimuli. Some regulatory considerations associated with auditory stimuli could include reducing the volume of sounds on EGMs, making them 'sound free' or ensuring that they play negative sounds when people lose on a spin. Losing sounds may also have a benefit for adults by making it clearer to determine losses which are arguably disguised as wins [48]. Other regulatory considerations could include ensuring that EGM rooms are located away from dining areas or venue entrances. While ultimately it is important to prevent children’s exposure, it is also important to recognise any potential unintended consequences associated with the annexing of EGM rooms including that hidden spaces may increase the risks associated with harmful gambling for adults [49].

Second is factors relating to how children *retain* knowledge about EGMs and those that work to *reinforce* positive perceptions. Children’s knowledge about EGMs were reinforced by the perceived behaviours of adults and the outcomes of these behaviours, and the perception that losses on EGMs enabled venues to give money to the community. Based on the behaviours of adults in their social networks, some children perceived that gambling on EGMs was a fun form of entertainment, that people win more than they lose, and that personal responsibility can protect people from harm. Messages about EGM use from government and industry focus heavily on personal responsibility [50, 51], and this study provides some indication that these messages may be reaching children. This is potentially problematic given that personal responsibility approaches appear to have had little impact on the prevention of harm from EGMs and may in fact lead to negative outcomes such as the stigmatisation of problem gamblers (Miller HE, Thomas S. The problem with ‘responsible gambling’: impact of government and industry discourses on feelings of felt and enacted stigma in people who experience problems with gambling. Addiction Research and Theory. Under submission). Further, it may contribute to misconceptions within the community about the structural factors that contribute to gambling harm, including the design features of EGMs [52]. Even though some children clearly held negative attitudes towards EGMs and understood that there were harms associated with these machines for individuals and families, these attitudes were softened for some children by a perception that people’s losses on EGMs resulted in community benefits via funding for community sports and other initiatives. While only a small percentage of losses from EGMs may be returned to the community via grants schemes in NSW (a m﻿inimum of﻿ 0.4% of anything over $1 million) [53], some children have a perception that ultimately losses will lead to good outcomes for them. We would not anticipate that children would be able to fully understand that the limited funding that goes back to the community is unlikely to outweigh the overall harms to the community. However, children who have positive experiences in venues, whose community activities benefit from venues or who hear positive stories about gambling from parents, also have little reason to believe that EGMs can cause significant harm for individuals and the community. They have no reason to think that EGMs may be addictive or that they may have a negative impact on individuals, their families and communities. This suggests that the promotion of community initiatives as part of corporate social responsibility initiatives may be successfully building positive brand images of clubs amongst community members, including children, which may ultimately soften perceptions of the harms associated with EGMs.

Third are factors that contribute to children *reproducing* behaviours associated with EGMs. A positive finding was that the vast majority of children perceived that allowing children to gamble would be irresponsible. This shows that there is an acknowledgement that these products are harmful for children. However, there were factors which influenced some children wanting to gamble on EGMs when they were older. Given that some children perceived that EGMs were fun 'games' which could be 'played', it is perhaps unsurprising that a few children perceived that because they won on the arcade games within the club, they would also have a positive and fun experience with EGMs. This calls into question the mixed messages that children may receive about gaming and gambling. While this is clearly an area for further research, we would recommend that there should be school-based education about EGMs, particularly in communities where there are high concentrations of gambling venues. While the Productivity Commission noted that there should be caution associated with education programmes about gambling [13], we would argue that education initiatives may be best modelled on successful programmes in alcohol and tobacco control which address information about the product, children’s product expectancies and the realities of product consumption [54, 55] rather than messages about individual responsibility. It is important that these programmes are developed independent of the gambling industry given that industry provision of education programmes to reduce gambling harm may come into conflict with their desire to maximise gambling revenue [56] and are carefully evaluated. Finally associated with reproducing behaviour is the issue associated with the role modelling of behaviour by significant adults. Children who knew adults who gambled on EGMs more commonly said that they would gamble on EGMs when they were older. Further research should explore whether parents understand the potential impact of their own EGM behaviours and how they talk about gambling may create positive perceptions of EGM use for children. This is particularly important when these behaviours occur within environments which are perceived to be family friendly, culturally valued and contain multiple fun activities for children.

Several limitations of this study should be noted. First, the study was exploratory with a small number of children and does not provide a comprehensive picture of all children’s perceptions and consumption intentions of EGMs. The study involved a small sample of families that attend local clubs in one geographical area. Given the limited prior research on this topic, future research should explore children’s perceptions of EGMs using larger and more diverse samples. This is important in identifying children’s relationships with EGMs in various geographic areas and whether children who are exposed to gambling venues have different knowledge and perceptions to children who do not visit such venues. The present study provides a starting point for further investigations into the impact of venues that promote themselves as family friendly but that also contain gambling products. Finally, this study specifically explored in-venue factors that influence children’s perception of EGMs. Future research should explore these factors in more detail in addition to investigating the role of other ancillary factors, including the influence of adult gambling behaviours.

## Conclusions

This study has shown that children are exposed to EGMs in venues through both visual and audio cues. This exposure and children’s awareness of adults EGM behaviours appeared to play a role in shaping children’s attitudes towards EGMs and future consumption intentions. Given the harm known to be associated with EGM use, this research provides a starting point for more comprehensive examinations of children’s exposure to gambling products within community venues, including the investigation of ancillary factors, which may influence children’s attitudes and future consumption intentions towards EGMs. We recommend that governments fund research into the attitudinal and behavioural impact of gambling venue layouts and product design to ensure public health policy is effective in preventing children’s exposure to adult gambling products within family-friendly venues.

## Abbreviations

EGMs: Electronic Gambling Machines
NSW: New South Wales
